# Molecular Epidemiology and Antimicrobial Resistance of Outbreaks of Klebsiella pneumoniae Clinical Mastitis in Chinese Dairy Farms

**DOI:** 10.1128/spectrum.02997-22

**Published:** 2022-11-14

**Authors:** Shaodong Fu, Chen Wen, Zhenglei Wang, Yawei Qiu, Yihao Zhang, Jiakun Zuo, Yuanyuan Xu, Xiangan Han, Zhenhua Luo, Wei Chen, Jinfeng Miao

**Affiliations:** a MOE Joint International Research Laboratory of Animal Health and Food Safety, Key Laboratory of Physiology and Biochemistry, College of Veterinary Medicine, Nanjing Agricultural University, Nanjing, China; b Shanghai Veterinary Research Institute, Chinese Academy of Agricultural Sciences, Shanghai, China; c School of Water, Energy and Environment, Cranfield University, Cranfield, United Kingdom; d Engineering Laboratory of Tarim Animal Diseases Diagnosis and Control, Xinjiang Production and Construction Crops, College of Animal Science, Tarim University, Tarim, China; Jilin University

**Keywords:** antimicrobial resistance, bovine clinical mastitis, *Klebsiella pneumoniae*, molecular epidemiology

## Abstract

Klebsiella pneumoniae is an opportunistic pathogen that causes serious infections in humans and animals. However, the availability of epidemiological information on clinical mastitis due to K. pneumoniae is limited. To acquire new information regarding K. pneumoniae mastitis, data were mined about K. pneumoniae strains on dairy cattle farms (farms A to H) in 7 Chinese provinces in 2021. Hypermucoviscous strains of K. pneumoniae were obtained by the string test. MICs of antimicrobial agents were determined via the broth microdilution method. Ten antimicrobial resistance genes and virulence genes were identified by PCR. The prevalence of K. pneumoniae was 35.91% (65/181), and 100% of the bacteria were sensitive to enrofloxacin. Nine antimicrobial resistance genes and virulence genes were identified and compared among farms. The hypermucoviscous phenotype was present in 94.44% of isolates from farm B, which may be a function of the *rmpA* virulence gene. Based on these data, the multidrug-resistant strains SD-14 and HB-21 were chosen and sequenced. Genotypes were assayed for K. pneumoniae isolates from different countries and different hosts using multilocus sequence typing (MLST). Ninety-four sequence types (STs) were found, and 6 STs present a risk for spreading in specific regions. Interestingly, ST43 was observed in bovine isolates for the first time. Our study partially reveals the current distribution characteristics of bovine K. pneumoniae in China and may provide a theoretical basis for the prevention and treatment of bovine K. pneumoniae mastitis.

**IMPORTANCE** *K. pneumonia* is ubiquitous in nature and infects a wide range of hosts, including animals, and humans. It is one of the leading inducements of clinical mastitis (CM) in dairy cows, a prevalent and costly disease that is predominantly associated with bacterial infection. In general, CM caused by Gram-negative bacteria is more difficult to cure than that associated with Gram-positive pathogens, with an average cost per case of 211.03 U.S. dollars (USD) for Gram-negative bacterial infections compared with 133.73 USD for Gram-positive bacterial CM cases. After Escherichia coli, K. pneumoniae is the second most common Gram-negative cause of bovine CM, but it is the most detrimental in terms of decreased milk yield, discarded milk, treatment costs, death, and culling. In view of the economic implications of K. pneumoniae infection in dairy farming, research into population structure and antibiotic resistance is particularly important.

## INTRODUCTION

Mastitis is one of the most common diseases in dairy herds worldwide. This disease directly affects animal welfare and results in decreased milk yield, increased cost of milk production, and reduced milk quality, leading to huge economic losses (1, 2). Previous mastitis control programs in dairy cows focused on the transmission of contagious mastitis pathogens, not environmental pathogens. More recently, contagious transmission of intramammary pathogens has been controlled through implementation of standard mastitis prevention programs. Environmental mastitis presents new challenges to modern dairy practices (2). Environmental mastitis is caused by a wide range of bacterial species, particularly Gram-negative bacteria, among which Klebsiella pneumoniae is a common cause of clinical disease (3, 4). Compared with Escherichia coli, K. pneumoniae may cause more severe clinical symptoms of mastitis and a stronger immune response (5). The concentrations of serum-binding globulin, interleukin 1 (IL-1), and IL-6 are upregulated more significantly as a result of K. pneumoniae infection (6). The resulting mastitis is difficult to diagnose and treat in its early stages. K. pneumoniae is present in soil, feed, drinking water, rumen contents, feces, bedding, and sewage. Bacteria from these sources, which are important reservoirs and carriers of K. pneumoniae, have direct contact with the udder (4). K. pneumoniae has also been found to be transmitted from infected to healthy cows, indicating a risk for lateral spread (7).

Increasing resistance of K. pneumoniae to the penicillin combination drugs is due to strains of K. pneumoniae (cKP strains) that are innately resistant to ampicillin and carboxybenzyl penicillin via production of *blaSHV-1*, which encodes beta-lactamase on chromosomes or a transferable plasmid (8, 9). Widespread use and misuse of antibiotics has led to the emergence of drug-resistant cKP, which is linked to an increased risk of side effects and resultant increased treatment costs. Once the multidrug-resistant cKP strains obtain the specific virulence factor of hypervirulent K. pneumoniae (hvKP) through horizontal gene transfer, new multidrug-resistant hvKP strains will appear, and K. pneumoniae will become more invasive (8). Carbapenem-producing K. pneumoniae has been isolated from humans in China, the United Kingdom, the United States, and other countries and regions (10). The antimicrobial resistance of K. pneumoniae from Chinese dairy products is unclear, warranting further study (11). K. pneumoniae in dairy cows often causes systemic infections that vary from mastitis to severe gastrointestinal disease (12). Four types of virulence genes, encoding K. pneumoniae fimbriae, capsules, lipopolysaccharides, and siderophores, have been identified (13). Other virulence factors, such as outer membrane proteins, porins, and ureases, are suspected to exist, but this has not been fully confirmed (14). The capsule virulence gene *rmpA* has been proven to regulate the overexpression of capsular substances of the highly mucous phenotype of K. pneumoniae with resultant high clinical invasiveness (15). It has also been demonstrated that *rmpA* and *kfuABC* are present in human clinical infections (16), While the genetic characteristics of virulence genes of K. pneumoniae in bovids have not been fully elucidated, the correlation to virulence among bovine and human isolates is important to explore (17).

Whole-genome sequencing (WGS) has developed rapidly. WGS was utilized to compare the antimicrobial resistance gene differences between bovine and human K. pneumoniae in the Middle East (18). Another study clarified the evolutionary relationship among isolates from different host sources during an epidemiological investigation of K. pneumoniae in bovine mastitis in the United States. The authors reported enzootic differences in metal resistance genes between bovine and human isolates (19). In China, genomic research on K. pneumoniae focuses mainly on human isolates, and data about bovine K. pneumoniae are lacking. Molecular typing is helpful in the study of the genetic and evolutionary relationships among individual bacteria and transmission between and within species. These data are of great value in the formulation of prophylactic measures. In the past, pulsed-field gel electrophoresis (PFGE) and repetitive element sequence-based PCR (rep-PCR) were often used to type K. pneumoniae based on the diversity of DNA fragments. These methods have the disadvantages of low reproducibility, poor stability, and quantitative difficulties (20). With the development of sequencing technology, these traditional typing methods are being replaced by WGS, which greatly improves the accuracy of typing and solves the problem of not being able to distinguish between closely related strains (21). Multilocus sequence typing (MLST) constructs a spectrum of 7 bacterial housekeeping alleles with good repeatability and high resolution (22). WGS is now the preferred method for microbial typing (23).

In the study described here, the molecular epidemiology and antimicrobial resistance of 65 strains of K. pneumoniae from Chinese dairy farms in various locations were analyzed. Multidrug-resistant strains were screened via WGS to determine their basic genetic characteristics. MLST was used to analyze and compare the genetic evolutionary relationship of K. pneumoniae strains prevalent in different regions of the world. Data from this study enrich the epidemiological understanding of K. pneumoniae clinical mastitis in China.

## RESULTS

### Incidence and phylogenetic diversity of K. pneumoniae on 8 dairy farms.

**FIG 1:**
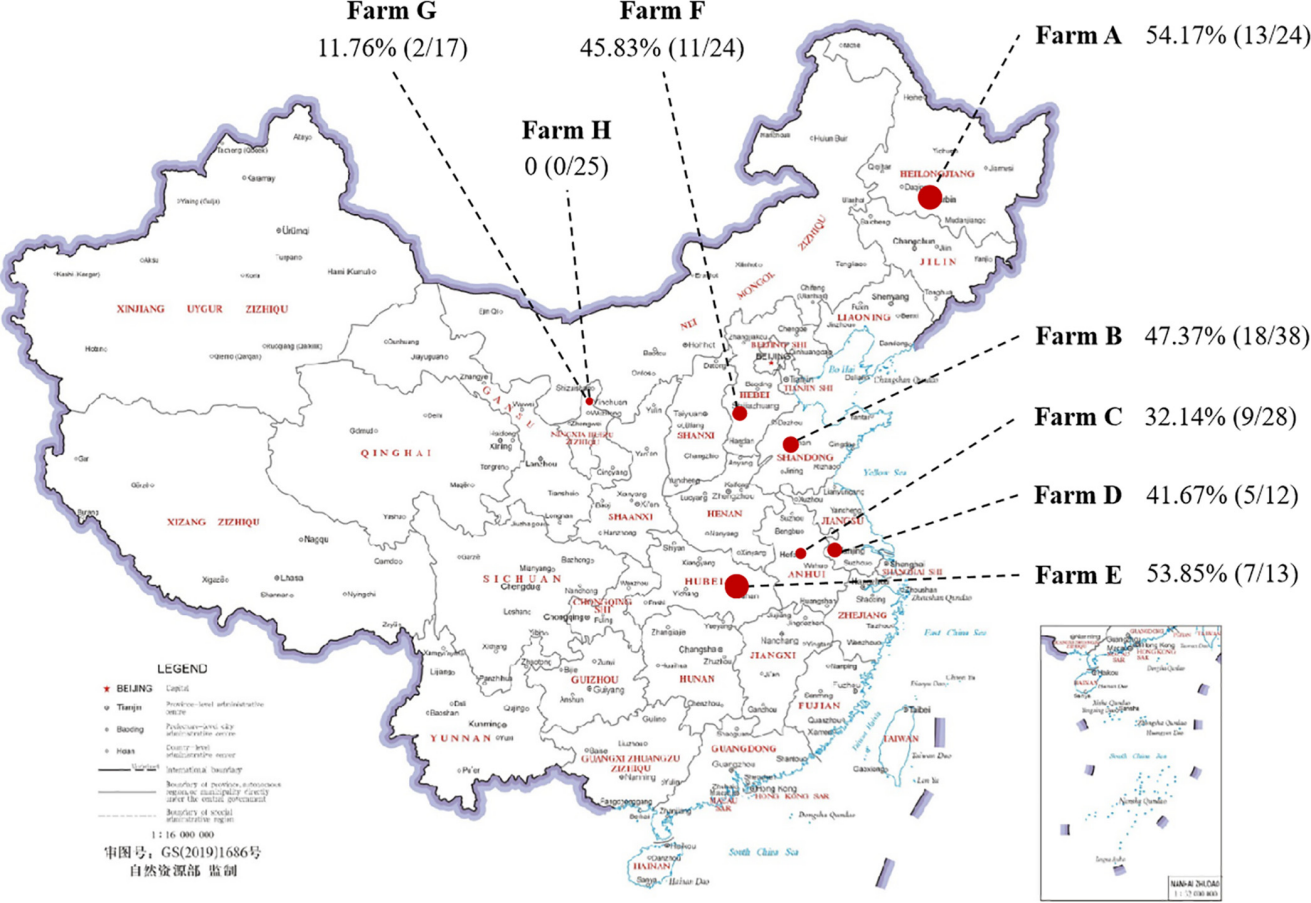
Percentage of K. pneumoniae in samples from cows with clinical mastitis in eight farms. The circle sizes indicate the detection rate of K. pneumoniae.

A total of 65 K. pneumoniae isolates were identified in 181 milk samples (35.91%) through biological identification, *khe* amplification, and 16S rRNA gene sequencing (Fig. 1; also, see Table S6 in the supplemental material). The samples collected from farm A (13/24, 54.17%) had the highest K. pneumoniae incidence. K. pneumoniae was undetectable in samples from farm H (0/25); K. pneumoniae incidence on farm G (2/17, 11.76%) was significantly lower than on farm A (13/24, 54.17%), farm B (18/38, 47.37%), farm E (7/13, 53.85%), and farm F (11/24, 45.83%) (*P* < 0.05). There were no significant differences in the K. pneumoniae incidence in milk from cows with clinical mastitis (CM) on farm G (2/17, 11.76%), farm C (9/28, 32.14%), and farm D (5/12, 41.67%). K. pneumoniae incidence in milk samples was found to be associated with the season (Table S7). The detection rate of K. pneumoniae increased with temperatures. Two major clades were present in the phylogenetic tree. Isolates from Jiangsu province had relatively close kinship with those in Shangdong province; others were more distantly related, although some were from the same district and farm (Fig. S1).

### Antimicrobial susceptibility testing and antimicrobial resistance genes.

**FIG 2:**
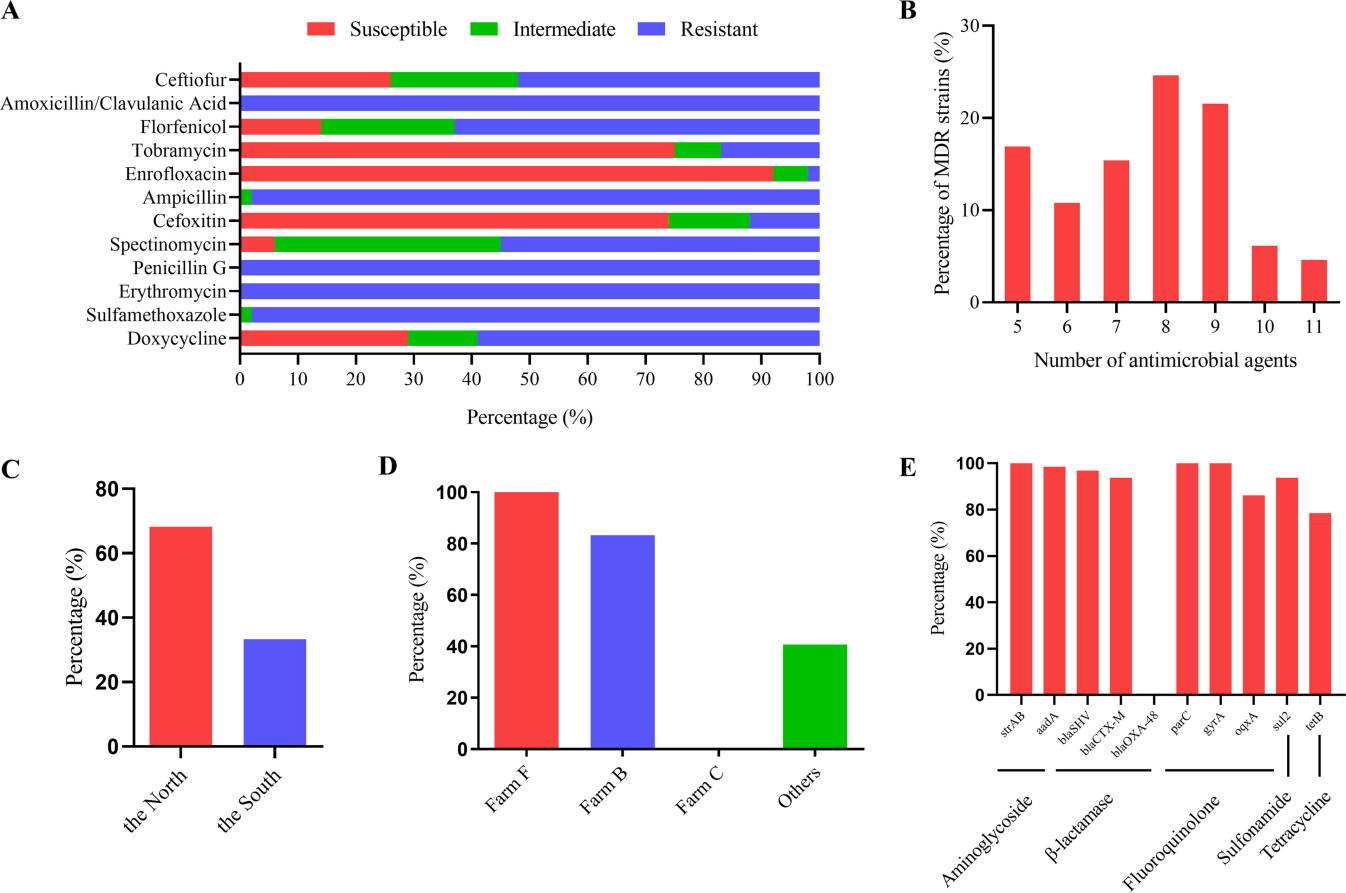
Bacterial resistance. (A) Resistance rate of K. pneumoniae to 12 antibiotics. (B) Percentages of MDR K. pneumoniae isolates resistant to different numbers of antibiotics. (C) Percentages of MDR K. pneumoniae isolates resistant to 8 or more antibiotics in the north and south regions of China. (D) Percentages of MDR K. pneumoniae isolates resistant to 8 or more antibiotics on different farms. (E) Rate of detection of resistance genes.

Susceptibility data for 12 antimicrobials are shown in Fig. 2A and Table S8. K. pneumoniae isolates were highly sensitive to enrofloxacin (92.31%), followed by tobramycin (75.38%) and cefoxitin (73.85%). However, K. pneumoniae was completely resistant to amoxicillin-clavulanic acid, penicillin G, and erythromycin and highly resistant to ampicillin (98.46%) and sulfamethoxazole (98.46%); the rates of resistance to ceftiofur, spectinomycin, florfenicol, and doxycycline were between 52.31 and 63.08%. The isolates were found to be resistant to at least 5 antibiotics. The dominant ones were resistant to 8 antibiotics (24.62%), followed by those resistant to 9 antibiotics (21.54%) (Fig. 2B). The rate of multidrug resistance (MDR) in K. pneumoniae for 8 or more antibiotics in northern farms (68.18%) was higher than that in southern farms (33.33%) (Fig. 2C). Moreover, the percentage of MDR K. pneumoniae isolates resistant to 8 or more antibiotics in farm F (100%) and farm B (83.33%) was higher than that in farm C (0%) and the other farms (40.74%) (Fig. 2D). Nine of 10 antimicrobial resistance genes were positively detected by PCR (Fig. 2E). The carbapenem resistance gene (*bla*_OXA-48_) was undetectable; the detection rates of the aminoglycoside resistance genes (*strAB*) and fluoroquinolone resistance genes (*parC* and *gyrA*) were 100%, and the remainder were between 78.46 and 98.46%. The detection rate of fluoroquinolone resistance genes (*parC*, *gyrA*, and *oqxA*) all exceeded 86.15%, which was different from the results of the antimicrobial susceptibility testing. All the K. pneumoniae isolates carried at least 6 resistance genes, and 60% of the isolates had 9 resistance genes (Fig. S2). The detection rate of fluoroquinolone resistance gene (*oqxA*) was only 27.27% in farm F, but it was nearly 100% in the others (Table 1). Notably, no significant difference was present in the carrier rate of resistance genes between farm C and the others (Table 1), which suggested that there may be differences between antimicrobial resistance phenotypes and resistance genes among K. pneumoniae isolates.

**TABLE 1:** Percentage of antimicrobial resistance genes of K. pneumoniae from seven farms

Type of antibiotic	Resistance gene	No. of positive samples/total (%) from farm:						
		A	B	C	D	E	F	G
Tetracyclines	*tetB*	8/13 (62)	13/18 (72)	7/9 (78)	5/5 (100)	7/7 (100)	9/11 (82)	2/2 (100)
Aminoglycosides	*aadA*	13/13 (100)	17/18 (94)	9/9 (100)	5/5 (100)	7/7 (100)	11/11 (100)	2/2 (100)
	*strAB*	13/13 (100)	18/18 (100)	9/9 (100)	5/5 (100)	7/7 (100)	11/11 (100)	2/2 (100)
β-Lactams	*bla*_OXA-48_	0/13 (0)	0/18 (0)	0/9 (0)	0/5 (0)	0/7 (0)	0/11 (0)	0/2 (0)
	*bla*_CTX-M_	11/13 (85)	18/18 (100)	9/9 (100)	5/5 (100)	5/7 (71)	11/11 (100)	2/2 (100)
	*bla*_SHV_	11/13 (85)	18/18 (100)	9/9 (100)	5/5 (100)	7/7 (100)	11/11 (100)	2/2 (100)
Fluoroquinolones	*oqxA*	12/13 (92)	18/18 (100)	9/9 (100)	5/5 (100)	7/7 (100)	3/11 (27)	2/2 (100)
	*parC*	13/13 (100)	18/18 (100)	9/9 (100)	5/5 (100)	7/7 (100)	11/11 (100)	2/2 (100)
	*gyrA*	13/13 (100)	18/18 (100)	9/9 (100)	5/5 (100)	7/7 (100)	11/11 (100)	2/2 (100)
Sulfonamides	*sul2*	11/13 (85)	18/18 (100)	8/9 (89)	5/5 (100)	6/7 (86)	11/11 (100)	2/2 (100)

### String test and virulence genes.

**FIG 3:**
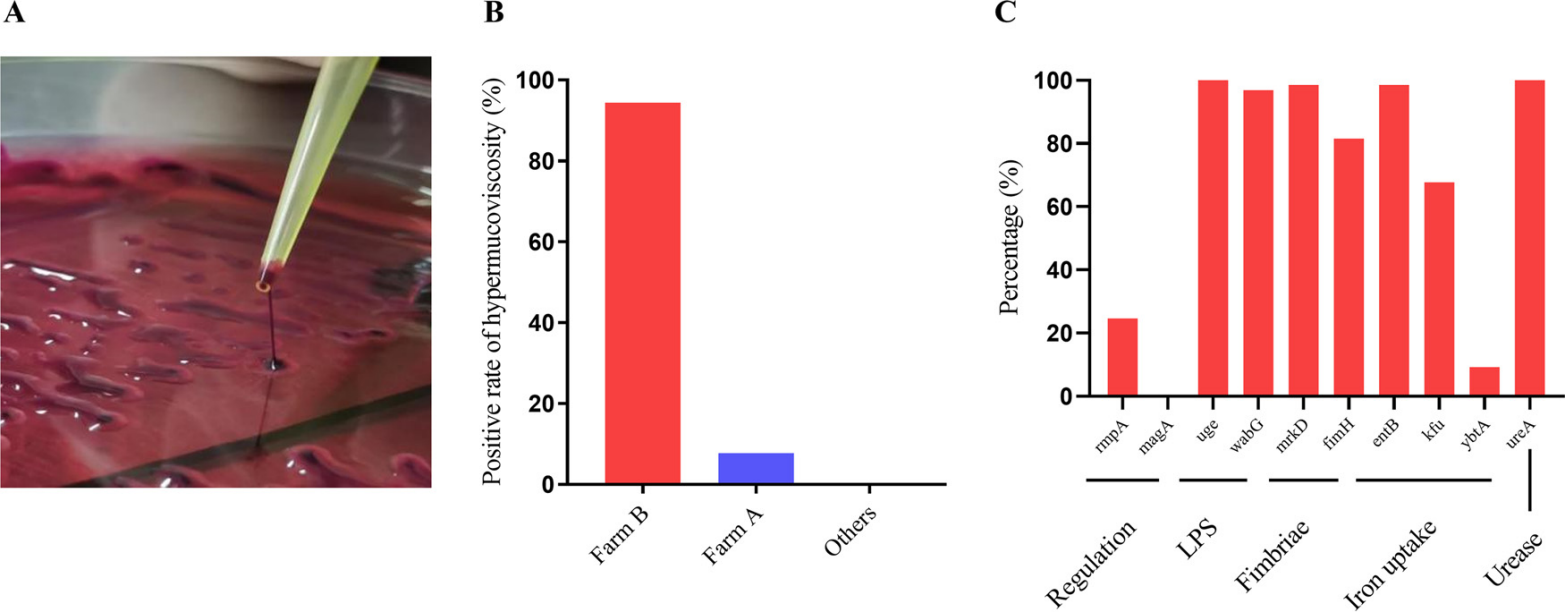
Results of the string test and bacterial virulence genes. (A) Colony morphology of hypermucoviscous K. pneumoniae. (B) Rate of hypermucoviscosity in K. pneumoniae isolates from different farms. (C) Rate of detection of virulence genes.

K. pneumoniae isolates grew well on eosin methylene blue (EMB) agar and formed characteristic pink to magenta colonies on MacConkey agar as a result of lactose fermentation. The purified bacterial isolates formed colonies that were round with convex surfaces and smooth margins and were viscous and moist. Eighteen K. pneumoniae isolates were determined to be hypermucoviscous by the string test (18/65, 27.69%) (Fig. 3A). The hypermucoviscous K. pneumoniae isolates were present only on farms A (1/13, 7.69%) and B (17/18, 94.44%) (Fig. 3B). Nine of 10 virulence genes were positively detected by PCR (Fig. 3C). The regulation-related gene (*magA*) was undetectable. The detection rates of a lipopolysaccharide-related gene (*uge*) and a urease-related gene (*ureA*) were 100%, and rates for other genes were between 9.23% and 98.46%. All the K. pneumoniae isolates carried at least 4 virulence genes, and 27.69% of the isolates were positive for 8 virulence genes (Fig. S3). The detection rates of a fimbria synthesis-related gene (*fimH*) and an iron uptake system gene (*kfu*) differed significantly among the farms. A iron uptake system gene (*ybtA*) was found only on farms B, D, and G (Table 2). A regulation-related gene (*rmpA*), which is associated with high virulence, was present only in hypermucoviscous K. pneumoniae in farm B (Table 2). There was a significant correlation between the *rmpA* genes and hypermucoviscosity.

**TABLE 2:** Percentage of virulence genes of K. pneumoniae from seven farms

Virulence factor	Related gene	No. of positive samples/total (%) from farm:						
		A	B	C	D	E	F	G
Regulation	*rmpA*	0/13 (0)	16/18 (89)	0/9 (0)	0/5 (0)	0/7 (0)	0/11 (0)	0/2 (0)
	*magA*	0/13 (0)	0/18 (0)	0/9 (0)	0/5 (0)	0/7 (0)	0/11 (0)	0/2 (0)
LPS	*wabG*	12/13 (92)	18/18 (100)	9/9 (100)	4/5 (80)	7/7 (100)	11/11 (100)	2/2 (100)
	*uge*	13/13 (100)	18/18 (100)	9/9 (100)	5/5 (100)	7/7 (100)	11/11 (100)	2/2 (100)
Fimbriae	*fimH*	11/13 (85)	18/18 (100)	9/9 (100)	5/5 (100)	6/7 (86)	2/11 (18)	2/2 (100)
	*mrkD*	13/13 (100)	18/18 (100)	8/9 (89)	5/5 (100)	7/7 (100)	11/11 (100)	2/2 (100)
Iron uptake	*entB*	12/13 (92)	18/18 (100)	9/9 (100)	5/5 (100)	7/7 (100)	11/11 (100)	2/2 (100)
	*ybtA*	0/13 (0)	1/18 (6)	0/9 (0)	4/5 (80)	0/7 (0)	0/11 (0)	1/2 (50)
	*kfu*	7/13 (54)	18/18 (100)	8/9 (89)	0/5 (0)	3/7 (43)	6/11 (55)	2/2 (100)
Urease	*ureA*	13/13 (100)	18/18 (100)	9/9 (100)	5/5 (100)	7/7 (100)	11/11 (100)	2/2 (100)

### Basic features of multidrug-resistant K. pneumoniae genome organizational structures.

The filtered sequencing data of K. pneumoniae SD-14 and HB-21 with high quality were obtained for subsequent assembly (Table S9). The genomes of SD-14 and HB-21 were found to be circular double-stranded DNA molecules of 5,326,114 bp (GC content, 57.43%) and 5,315,618 bp (GC content, 57.36%), respectively. The DNA replication origins of SD-14 (Fig. S4) and HB-21 (Fig. S5) were predicted by the GC skews. Genomic islands (GIs) usually contribute to lateral gene transfer and bacterial evolution. We identified 11 GIs in SD-14 and 12 GIs in HB-21 (Tables S10 and S11). There was no clustered regularly interspaced short palindromic repeat sequences (CRISPRs) in the genomes of SD-14 and HB-21. Gene ontology (GO) analysis had the highest enrichment ratio in the plasma membrane, and it exhibited high metal ion binding activity in terms of molecular function (Fig. S6). KEGG pathway enrichment analysis had the highest enrichment ratio in the carbohydrate metabolism pathway (Fig. S7).

### Multilocus sequence typing and phylogenetic comparison of K. pneumoniae among different sources and countries.

**FIG 4:**
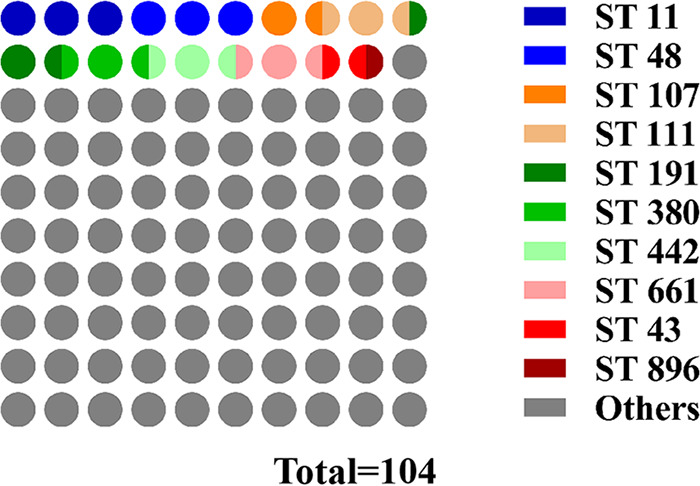
Proportions of 94 STs in 104 strains of K. pneumoniae. Different colors indicate percentages of the indicated STs in 104 K. pneumoniae isolates.

**FIG 5:**
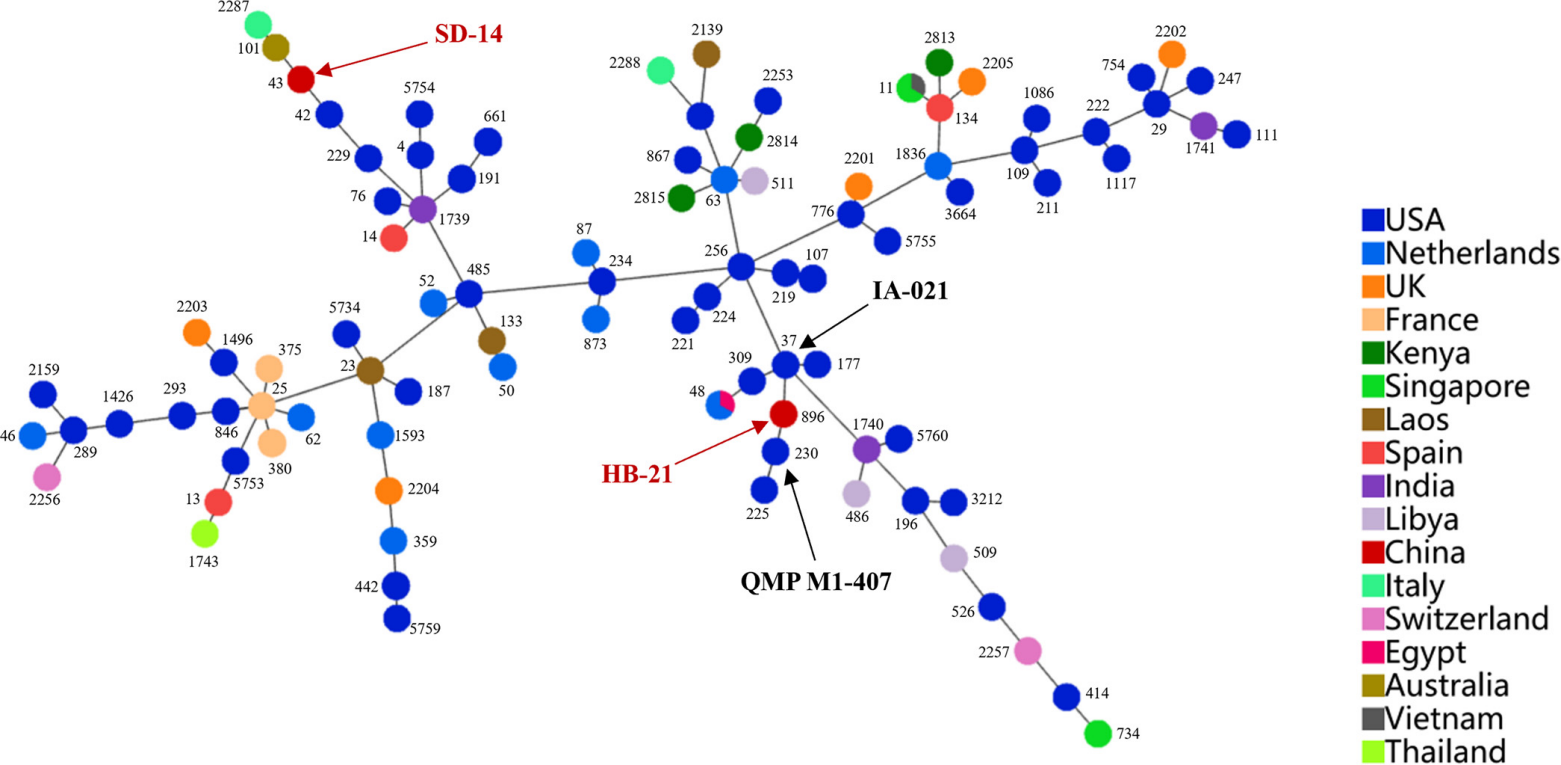
Burst map of 104 K. pneumoniae STs. Each circle represents one sequence type labeled with ST name; sizes indicate the total number of strains. Branch lengths indicate phylogenetic distances between STs. Different colors indicate percentages of certain STs in different countries.

Based on alleles of 7 conserved MLST genes (*gapA*, *infB*, *mdh*, *pgi*, *phoE*, *rpoB*, and *tonB*), K. pneumoniae SD-14 and HB-21 were classified as ST43 and ST896, respectively, which are not new sequence types (STs). Notably, ST43 was first observed in bovine K. pneumoniae isolates in this study, and there were no indications that ST43 was related to carbapenem resistance genes. These data differ from those from previous studies with humans. All 104 strains of K. pneumoniae were identified as 94 different STs, among which ST11 (3/104, 2.88%) and ST48 (3/104, 2.88%) accounted for the largest proportion, followed by ST107 (2/104, 1.92%), ST111 (2/104, 1.92%), ST191 (2/104, 1.92%), ST380 (2/104, 1.92%), ST442 (2/104, 1.92%), and ST661 (2/104, 1.92%). The predominant sequence types ST107, ST111, ST191, ST442, and ST661 have previously been identified only in the United States, and ST380 has been identified only in France. ST48 is present in both human and bovine isolates. The other 86 STs were all detected in only one strain; however, they comprised 82.69% (86/104) of the whole population, which confirmed the high genomic diversity (Fig. 4 and Table S12). eBURST analyses demonstrate the evolutionary relationship among 94 STs (Fig. 5). SD-14 (ST43) and HB-21 (ST896) were classified into 2 STs which were distant from each other. There was only the difference of a *tonB* gene between HB-21 (ST896) and IA-021 (ST37), There was only a different *infB* gene between HB-21 (ST896) and QMP M1-407 (ST230). Except for the France strains, the strains from other countries had distant genetic relationships. The distribution of the strains from different countries and hosts had no regular pattern, which also confirmed high genomic diversity.

## DISCUSSION

A plethora of studies have shown that 20% of bovine mastitis is due to K. pneumoniae infection (24). Because most studies have focused on human isolates, the epidemiology and pathogenic mechanisms of bovine K. pneumoniae are not fully understood, which hampers clinical treatment. The current study assayed 181 clinical mastitic milk samples from 9 large-scale dairy farms in 7 Chinese provinces from July to October 2021. Suspected K. pneumoniae colonies were purified by plate streaking and stored after identification of *khe* gene and 16S rRNA gene sequences. Sixty-five strains of K. pneumoniae were isolated, with a total isolation rate of 35.9%. The detection rate of K. pneumoniae in Ningxia (farm G and farm H) is significantly lower than that in other regions. The detection rate of K. pneumoniae in July and August was significantly higher than in September and October. Thus, in summer, with higher temperatures and a more humid environment, K. pneumoniae colonization may have increased, resulting in a higher infection rate. Bovine heat stress may increase susceptibility to pathogenic bacteria due to downregulation of immune responses. There is a paucity of epidemiologic data regarding milk-derived K. pneumoniae. Song et al. (25) conducted an epidemiological mastitis survey in 3 large-scale dairy farms from January 2018 to December 2019 in Shandong, Hebei, and Heilongjiang provinces. Due to a large number of collections and long detection time, the detection rates of K. pneumoniae were only 3.0% and 2.8% in 2018 and 2019, respectively. In addition, in order to explore potential transmission and reservoirs for mastitis caused by K. pneumoniae, Cheng et al. (26) analyzed milk samples from 2 large Chinese dairy farms (farm A in January 2019 and farm B in May 2019), and the isolation rates from clinical mastitis were 34% and 42% for farm A and farm B, respectively, which further illustrated the effect of temperature on the detection rate. The previous observations combined with our data indicate that it is likely that the infection rate of K. pneumoniae in Chinese dairy cows has risen over time. Dairy farms should strengthen prophylactic and therapeutic modalities to reduce intercurrent disease and strictly implement daily feeding management and production measures to alleviate and control K. pneumoniae disease.

All of the 65 K. pneumoniae strains were most sensitive to enrofloxacin, indicating that enrofloxacin is a preferred antibiotic for the treatment of K. pneumoniae mastitis. The isolates were generally resistant to 12 antibiotics, among which the rates of resistance to amoxicillin-clavulanic acid and ampicillin were 100% and 98.46%, respectively. These data may be related to the natural existence of β-lactamase in K. pneumoniae (8). Saini et al. (27) reported that the rates of resistance to sulfamethoxazole and doxycycline of the K. pneumoniae isolates from Canadian dairy farms were 11.7% and 18.6%, respectively. Data from the current study showed that the rates of resistance to sulfamethoxazole and doxycycline were 98.46% and 58.46%. This disparity may be due to differences in antibiotics use among countries and to the spatial distribution of the isolates. The resistance of Chinese isolates from the south was weaker than that from the north, which may be a function of the types and frequency of antibiotic use in the north. The resistance of isolates from Hebei Province (farm F) was the strongest, while that of isolates from Heilongjiang Province (farm A) was significantly weaker than that in other regions, indicating that there are differences in the resistance of milk-derived K. pneumoniae isolates from different regions of China.

The prevalences of the fluoroquinolone resistance genes *parC*, *gyrA*, and *oqxA* were 100%, 100%, and 86.2%, respectively, which are inconsistent with the rates of resistance to sulfamethoxazole (98.46%) and enrofloxacin (1.54%), suggesting that these 3 genes may mediate the resistance phenotype of the isolates to sulfamethoxazole rather than enrofloxacin. These phenomena may be a function of undetected antimicrobial resistance genes or unidentified mechanisms of resistance. Carbapenemase-producing K. pneumoniae is a challenge to current clinical treatment (28), and *bla*_OXA-48_ is an important gene encoding carbapenemase. *bla*_OXA-48_ was absent in all strains of K. pneumoniae in this study. Reports of the *bla*_OXA-48_ gene in bovine isolates are rare, and the epidemic differences between human and bovine isolates has not been studied. The coding sites of *blaSHV* are often similar to those of aminoglycoside, fluoroquinolone, and sulfonamide resistance genes, which may be responsible for multidrug resistance. In the current study, the detection rate of *bla*_SHV_ was 96.92%, which may reflect the high rates of resistance of the isolates to sulfamethoxazole and spectinomycin. An *Enterobacteriaceae* isolate carrying *bla*_CTX-M_ was first reported in the United States in 2003 (29), and it is most commonly distributed in the United States, China, Japan, France, and Germany (30), which is consistent with data from our study. The differences in virulence genes among isolates was mainly expressed in *rmpA* and *ybtA*. *rmpA* and *magA*, which have been linked to the formation of hypermucoviscosity, are related to the high pathogenicity of hvKP (31). It is of interest that *rmpA* was present only in isolates with hypermucoviscosity in Shandong Province (farm B), suggesting that the prevalent K. pneumoniae isolates in Shandong may have higher pathogenicity. Whether these isolates are hvKP strains needs to be verified in combination with clinical trials. The iron uptake system gene *ybtA* was present only in Shangdong Province (farm B), Jiangsu Province (farm D), and Ningxia Province (farm G), which suggests that *ybtA* has regional epizootic characteristics in China. The virulence gene for iron uptake, *kfu*, is related to the invasiveness of K. pneumoniae in humans (32). Subsequent studies found that *kfu* was also related to clinical mastitis infection in cows (33). The rate of detection of *kfu* in this study reached 67.69%, which is consistent with the above conclusion.

The six leading milk-producing provinces in China are Hebei, Inner Mongolia, Shandong, Heilongjiang, Shanxi, and Henan provinces, which contribute more than 50% of the national production. Furthermore, on the basis of the incidence rate of mastitis, the isolation rate of K. pneumoniae in clinical mastitis, and the results of the string test as well as detection of virulence and drug resistance, representative MDR strains SD-14 and HB-21 were selected to delve further into the genetic characteristics. CRISPR is a defense system for bacteria to resist foreign DNA. Previous research showed that about 60% of bacteria lack a CRISPR system, and it is common to see the acquisition and loss of the system (34). Rollie et al. (35) found that this phenomenon may be related to the host autoimmune response caused by the CRISPR system. Bacteria that lose the CRISPR system may avoid the damage caused by their own targeted immune response and have a survival advantage. There was no CRISPR sequence found in SD-14 and HB-21 genomes, indicating that milk-derived K. pneumoniae has evolved its own protective measures against environmental pressure. In addition, GO analysis revealed that the genes carried by SD-14 and HB-21 were closely related to metal ion binding activity, which has been proved to be significantly related to the virulence of K. pneumoniae (36). The metal ion binding activity of hvKP is generally higher than that of cKP, suggesting that SD-14 and HB-21 may have high pathogenicity, which is worthy of further study. SD-14 and HB-21 were identified as ST43 and ST896, respectively. Notably, ST43 was first observed in bovine K. pneumoniae isolates in this study. Earlier studies on ST43 K. pneumoniae in humans showed that it was characterized by carbapenem resistance genes (37). However, the carbapenem resistance gene *bla*_OXA-48_ was undetectable in SD-14 in the current study, suggesting significant differences between bovine and human isolates. This phenomenon may be directly related to the use of clinical drugs. Conversely, K. pneumoniae in milk samples may originate from the hands of the milker, which suggests that the bacterial infection and resultant mastitis may be zoonotic. The results of eBURST analysis (ST burst map) that only the *tonB* gene differed between HB-21 (ST896) and IA-021 (ST37) and only the *infB* gene differed between HB-21 (ST896) and QMP M1-407 (ST230), indicating that HB-21 is closely related to the prevalent K. pneumoniae strain in the United States. Moreover, there is high genomic diversity of K. pneumoniae from various regions of the world.

This study has limitations, including, the large south-north distance, regional climate differences, and varying husbandry protocols, including antibiotic usage. A larger sample size would aid in data verification. In summary, the resistance of K. pneumoniae to clinically common antibiotics is present in the regions of China studied. Multidrug resistance of isolated strains with the hypermucoviscosity phenotype from Shandong Province is severe. MDR strain HB-21 from Hebei Province is very similar to the K. pneumoniae strain in the United States. Our data provide the groundwork for developing prevention and treatment modalities for bovine K. pneumoniae mastitis.

## MATERIALS AND METHODS

### Farm distribution and sample collection.

Samples were collected from 8 commercial farms (farms A to H) in 7 provinces, including Heilongjiang, Hebei, Anhui, Shandong, Ningxia, Hubei, and Jiangsu provinces, from July to October 2021. Lactating cows from 9 farms were milked 3 times/day in milking parlors. The incidence of mastitis for each month was calculated for 6 farms (Table S1). Clinical mastitis was diagnosed by at least 1 of the following symptoms: elevated udder temperature, clots in the milk, and swelling of the udder (38, 39). The clinical signs were augmented via the California mastitis test (CMT). The farms used a variety of antibacterial agents, including ceftiofur, amoxicillin-clavulanic acid, kanamycin, and penicillin G, for regular prophylaxis and treatment. Table S1 includes 181 milk samples from clinically mastitic cows from farms A to H. Briefly, milk samples were collected from lactating cows by the milkers after teat disinfection, and the first 3 streams of milk were discarded. Fifty-milliliter sterile centrifuge tubes were used to collect milk samples. Samples were stored at 4°C and transported to the laboratory within 24 h for bacterial culturing and identification.

### Isolation and identification of K. pneumoniae.

Milk samples were centrifuged for 15 min (4,235 × *g*, 4°C); the bacteria were precipitated, and supernatants were discarded. Approximately 3 mL of phosphate-buffered saline (PBS) was added to resuspend the bacteria. The resuspended samples were inoculated in 10 mL EC broth medium (Haibo Biotech, Qingdao, China) and incubated at 37°C for 18 to 24 h. A loopful of broth culture was streaked onto EMB agar (Yifeixue Biotech, Nanjing, China) and incubated at 37°C for 12 to 18 h. A single pink, smooth, and slimy suspected colony was harvested and purified on EMB agar. Purified bacteria underwent biochemical identification and PCR testing. Gram staining was performed with a Gram stain kit (Solarbio Technology, Beijing, China). Further identification and confirmation of the isolates were carried out by PCR of *khe* (GenBank accession no. CP035202.1) and 16S rRNA genes (26, 40). The 16S rRNA genes of the isolates were amplified using the 16S universal primers 27F (5′-AGAGTTTGATCCTGGCTCAG-3′) and 1525R (5′-AAGGAGGTGATCCAGCCGCA-3′). The PCR assay was carried out in a 25-μL reaction mixture containing 12.5 μL of 2× Accurate *Taq* master mix (Accurate Biotechnology (Human) Co., Ltd., Changsha, China), composed of 1 μL of each primer, 2 μL of template DNA, and 8.5 μL of sterile water. PCR conditions were as follows: initial denaturation at 94°C for 4 min, denaturation at 94°C for 30 s, annealing at 55°C for 30 s, extension at 72°C for 30 s (35 cycles), and finally extension at 72°C for 10 min. PCR products were electrophoresed on a 1% (wt/vol) agarose gel with TS-GelRed (Tsingke Biotech, Nanjing, China) and visualized under UV light. Positive clones were sequenced by the Tsingke Biotechnology Corporation, Ltd. (Nanjing, China). K. pneumoniae CMCC 46117 (*khe* gene positive) was used as the control strain. To clarify the genetic relatedness among the strains, a phylogenetic tree was constructed according to the neighbor-joining method using MEGA7.0.

### DNA extraction.

Genomic DNA of K. pneumoniae was extracted by a boiling method. Briefly, 1.5 mL suspended plaque samples were centrifuged for 15 min (13,523 × *g*, 4°C). The supernatants were discarded, and the sediments were resuspended in 1 mL of PBS; these steps were repeated 3 times. The resuspended samples were incubated for 15 min at 99°C. The supernatants were cooled to −20°C immediately after centrifugation (15 min, 13,523 × *g*, 4°C) for PCR testing.

### Antimicrobial susceptibility testing.

Twelve broad-spectrum antimicrobials commonly used on dairy farms were employed to detect the susceptibility of K. pneumoniae isolates by the broth microdilution method recommended by the Clinical and Laboratory Standardization Institute (CLSI), including β-lactam–β-lactamase inhibitor combinations of amoxicillin-clavulanic acid (AMC), ampicillin (AMP), penicillin G (PG), and ceftiofur (EFT); the fluoroquinolones enrofloxacin (ENR) and sulfamethoxazole (SXT); the aminoglycosides spectinomycin (SH) and tobramycin (TOB); the cephamycin cefoxitin (CXT); the chloramphenicol florfenicol (FFC); the macrolide erythromycin (ERM); tetracyclines; and doxycycline (DOX). Results were interpreted according to the CLSI M100-S30 standards (Table S2). The phenotypic resistance patterns were categorized as MDR as previously described by Magiorakos et al. (41). The quality control strain was E. coli ATCC 25922.

### Detection of antimicrobial resistance genes and virulence genes.

Several genes encoding resistance to β-lactams (*bla*_SHV_, *bla*_CTX−M_, and *bla*_OXA-48_), aminoglycosides (*aadA* and *strAB*), fluoroquinolones (*parC*, *gyrA*, and *oqxA*), sulfonamides (*sul2*), and tetracyclines (*tetB*) were analyzed by PCR (Table S3). All 65 K. pneumoniae isolates were evaluated for 10 virulence genes, including capsular polysaccharide synthesis and synthesis regulation-related genes (*rmpA* and *magA*), lipopolysaccharide-related genes (*uge* and *wabG*), fimbria synthesis-related genes (*mrkD* and *fimH*), iron uptake system genes (*entB*, *kfu*, and *ybtA*), and a urease-related gene (*ureA*), by PCR (Table S4). Primer synthesis was conducted by the Tsingke Biotechnology Corporation Ltd. (Nanjing, China).

### String test of K. pneumoniae.

Like all capsulated bacteria, K. pneumoniae produces mucoid colonies in a nutritive medium. This mucoid phenotype differs from a hypermucoviscous phenotype in that hypermucoviscosity is defined by the formation of a ≥5-mm viscous filament when a K. pneumoniae colony is stretched with a loop on an agar plate (42). Purified bacterial samples were inoculated in 2 mL lysogeny broth medium (Haibo Biotech, Qingdao, China) and incubated at 37°C for 18 to 24 h. A loopful of broth culture was streaked onto MacConkey agar (Haibo Biotech, Qingdao, China) and incubated at 37°C for 18 h. The colony was stretched by a loop, which should be repeated three times, and the results of the test were recorded.

### Sequencing and genomic assembly.

Representative multidrug-resistant strains SD-14 and HB-21 were streaked onto MacConkey agar plates and incubated overnight at 37°C. Single colonies were inoculated into lysogeny broth medium for another 12 h with shaking at 180 rpm/min. Two milliliters of bacterial culture broth was harvested for genomic DNA purification following the procedure of the TIANamp bacteria DNA kit (Tiangen Biotech, Beijing, China). DNA yields of the K. pneumoniae strains were measured at a wavelength of 260/280 nm by a NanoDrop Lite spectrophotometer.

Genomes of K. pneumoniae strains SD-14 and HB-21 were sequenced on the Illumina NovaSeq 6000 sequencing platform and Nanopore promethION sequencing platform. Sequencing was conducted by Allwegene Gene Technology Co., Ltd. (Nanjing, China). Sequencing data were filtered and counted in order to ensure the quality of subsequent assembly analysis. The off-line original data of Nanopore sequencing were in the form of a fast5 file. Base calling was used to convert the data to fastq format by GUPPY 4.4.2. The data were filtered to obtain effective data (*Q* ≥ 7) for subsequent assembly analysis. Original Illumina data were filtered by SOAPnuke (version 2.1.2) (43). A Unicycler 0.4.9 (44) was used to assemble the high-accuracy Illumina data (*Q*_30_ > 85%) to obtain a high-quality bacterial genome skeleton (contig), and the high-quality contig was spliced into completed genome maps with Nanopore data. Finally, Pilon 1.23 (45) was used to further correct the assembled genome by using the Illumina data to obtain a more accurate genome. Prokka 1.12 (46) and Prodigal v2.6.3 (47) were used to predict the coding genes, Aragorn v1.2.38 (48) to predict tRNA, RNAmmer 1.2 (49) to predict rRNA, and Infrared 1.1 (50) to predict miscellaneous RNA. MinCED 0.4.2 was used to predict CRISPR sequences. IslandViewer 4 was used to predict the gene islands. Finally, the predicted gene sequences of SD-14 and HB-21 were compared with GO and KEGG function databases by BLAST 2.5.0+.

### Multilocus sequence typing.

STs were assigned to K. pneumoniae SD-14 and HB-21 genomes according to 7 highly conserved housekeeping gene sequences from the Institut Pasteur MLST database (https://bigsdb.pasteur.fr/): *gapA* (glyceraldehyde-3-phosphate dehydrogenase A), *infB* (translation initiation factor IF-2), *mdh* (malate dehydrogenase), *pgi* (glucose-6-phosphate isomerase), *phoE* (phosphoporin), *rpoB* (RNA polymerase subunit beta), and *tonB* (TonB system transport protein).

Information on K. pneumoniae isolates from different countries and different hosts was downloaded from the PubMLST database. A burst map of ST was generated by PHYLOViZ (51).

### Statistical analyses.

SPSS statistics version 26.0 and GraphPad Prism version 8.0 software were used for statistical analyses. The chi-squared test (χ^2^) was used to compare the statistical significance between the different groups. A *P* value of <0.05 is considered significant, and a *P* value of <0.01 is considered extremely significant.

### Data availability.

The whole-genome sequences of K. pneumoniae strains SD-14 and HB-21 have been deposited in NCBI’s Sequence Read Archive and are accessible through BioProject no. PRJNA820152 (isolate accession no. SAMN26982150 and SAMN26982151). Details of 102 other publicly available K. pneumoniae whole-genome sequences are listed in Table S5.
