# Supplementary material for: Molecular Epidemiology and Antimicrobial Resistance of Outbreaks of Klebsiella pneumoniae Clinical Mastitis in Chinese Dairy Farms
Source: Microbiol Spectr. 2022 Nov 14;10(6):e02997-22. doi: 10.1128/spectrum.02997-22 (PMC9769803; doi:10.1128/spectrum.02997-22)
Supplement: Supplemental file 1 — Supplemental material. Download spectrum.02997-22-s0001.pdf, PDF file, 0.9 MB [file spectrum.02997-22-s0001.pdf]

Supplementary Material

Table S1 Specific source information of milk samples.

Dairy farm	Location	Incidence of mastitis (%)	Sample quantity
Farm A	Heilongjiang, China	2.82	24
Farm B	Shangdong, China	2.13	38
Farm C	Anhui, China	Unknown	28
Farm D	Jiangsu, China	Unknown	12
Farm E	Hubei, China	2.26	13
Farm F	Hebei, China	3.00	24
Farm G	Ningxia, China	2.20	17
Farm H	Ningxia, China	1.74	25

Table S2 Breakpoints of 12 Antibiotics for *K. pneumoniae*.

Antibiotic	Breakpoints		
	S	I	R
Penicillin G	≤16		≥32
Ampicillin	≤8	16	≥32
Amoxicillion/clavulanic acid	≤4	8	≥16
Sodium Ceftiofur	≤1	2	≥4
Enrofloxacin	≤4	8	≥16
Sulfamethoxazole	≤256		≥512
Spectinomycin	≤16	32	≥64
Tobramycin	≤4	8	≥16
Cefoxitin	≤8	6	≥32
Florfenicol	≤4		≥16
Erythromycin	≤16		≥32
Doxycycline	≤4	8	≥16

Table S3 Primers for antimicrobial resistance genes and product size of *K. pneumoniae*.

Genes	Sequence (5'-3')	Amplicon size (bp)	Annealing temperature (℃)
bla$_{OXA-48}$	GCGTGGTTAAGGATGAACAC<br>CATCAAGTTCAACCCAACCG	438	52
bla$_{SHV}$	GCCTTTATCGGCCTTCACTCAAG<br>TTAGCGTTGCCAGTGCTCGATCA	898	55
bla$_{CTX-M}$	AACCGTCACGCTGTTGTTAG<br>TTGAGGCGTGGTGAAGTAAG	766	52
oqxA	CTCGGCGCGATGATGCT<br>CCACTCTTCACGGGAGACGA	392	55
parC	CTGAATGCCAGCGCCAAATT<br>TGCGGTGGAATATCGGTCGC	382	54
gyrA	CGCGTACTATACGCCATGAACGTA<br>ACCGTTGATCACTTCGGTCAGG	420	55
sul2	CGGCATCGTCAACATAACC<br>GTGTGCGGATGAAGTCAG	722	50
tetB	CTCAGTATTCCAAGCCTTTG<br>CTAAGCACTTGTCTCCTGTT	416	57
strAB	TATCTGCGATTGGACCCTCTG<br>CATTGCTCATCATTTGATCGGCT	538	55
aadA	GCAGCGCAATGACATTCTTG<br>ATCCTTCGGCGCGATTTTG	282	60

Table S4 Primers for virulence genes and product size of *K. pneumoniae*.

Genes	Sequence (5'-3')	Amplicon size (bp)	Annealing temperature (℃)
wabG	ACCATCGGCCATTTGATAGA<br>CGGACTGGCAGATCCATATC	683	50
uge	TCTTCACGCCTTCCTTCACT<br>GATCATCCGGTCTCCCTGTA	534	51
fimH	TGCTGCTGGGCTGGTCGATG<br>GGGAGGGTGACGGTGACATC	550	57
mrkD	AAGCTATCGCTGTACTTCCGGCA<br>GGCGTTGGCGCTCAGATAGG	340	57
entB	GTCAACTGGGCCTTTGAGCCGTC<br>TATGGGCGTAAACGCCGGTGAT	400	57
kfu	ATAGTAGGCGAGCACCGAGA<br>AGAACCTTCCTCGCTGAACA	530	57
ureA	GCTGACTTAAGAGAACGTTATG<br>GATCATGGCGCTACCT(C/T)A	337	50
ybtA	ATGACGGAGTCACCGCAAAC<br>TTACATCACGCGTTTAAAGG	960	55
rmpA	TACATATGAAGGAGTAGTTAAT<br>GAGCCATCTTTCATCAAC	505	48
magA	GGTGCTCTTTACATCATTGC<br>GCAATGGCCATTTGCGTTAG	1238	49

Table S5 Specific background information of 102 *K. pneumoniae* strains.

Isolate ID	Strain	Country	Year	Source	Accession No.
M1	WI-01	USA	2014	Bovine	SAMN13523781
M2	MN-01	USA	2014	Bovine	SAMN13523782
M3	MN-02	USA	2014	Bovine	SAMN13523783
M4	WI-02	USA	2014	Bovine	SAMN13523786
M5	FL-01	USA	2014	Bovine	SAMN13523787
M6	FL-02	USA	2014	Bovine	SAMN13523789
M7	VT-01	USA	2014	Bovine	SAMN13523791
M8	FL-03	USA	2014	Bovine	SAMN13523792
M9	FL-04	USA	2015	Bovine	SAMN13523793
M10	CT-01	USA	2015	Bovine	SAMN13523795
M11	PA-01	USA	2015	Bovine	SAMN13523803
M12	PA-02	USA	2015	Bovine	SAMN13523804
M13	NC-01	USA	2015	Bovine	SAMN13523807
M14	MN-11	USA	2016	Bovine	SAMN13523815
M15	WA-01	USA	2016	Bovine	SAMN13523816
M16	MN-12	USA	2016	Bovine	SAMN13523818
M17	WI-08	USA	2016	Bovine	SAMN13523819
M18	WI-09	USA	2016	Bovine	SAMN13523820
M19	WI-10	USA	2016	Bovine	SAMN13523821
M20	WI-11	USA	2016	Bovine	SAMN13523822
M21	NY-05	USA	2016	Bovine	SAMN13523823
M22	NY-06	USA	2016	Bovine	SAMN13523824
M23	NY-07	USA	2016	Bovine	SAMN13523825
M24	NC-02	USA	2017	Bovine	SAMN13523829
M25	CT-02	USA	2017	Bovine	SAMN13523831
M26	CT-03	USA	2017	Bovine	SAMN13523837
M27	CT-04	USA	2017	Bovine	SAMN13523840
M28	NY-10	USA	2017	Bovine	SAMN13523841
M29	NY-11	USA	2017	Bovine	SAMN13523842
M30	NY-12	USA	2017	Bovine	SAMN13523844
M31	MN-20	USA	2018	Bovine	SAMN13523848
M32	CA-01	USA	2018	Bovine	SAMN13523849
M33	IA-001	USA	2015	Bovine	SAMN13523850
M34	IA-004	USA	2015	Bovine	SAMN13523853
M35	IA-018	USA	2015	Bovine	SAMN13523867
M36	IA-020	USA	2015	Bovine	SAMN13523869
M37	IA-021	USA	2015	Bovine	SAMN13523870
M38	IA-022	USA	2015	Bovine	SAMN13523871
M39	IA-059	USA	2015	Bovine	SAMN13523908
M40	K3	Egypt	NA	Bovine	SRR13933220
M41	SB2722	Netherlands	2009	Bovine	Unknown

M42	SB2726	Netherlands	2009	Bovine	Unknown
H1	SKP000534	USA	2008	Human	ERR025515
H2	SKP000795	USA	2006	Human	ERR025140
H3	SKP000800	USA	2006	Human	ERR025986
H4	SKP000802	USA	2007	Human	ERR025988
H5	SKP000803	USA	2007	Human	ERR025989
H6	KP01	Netherlands	2016	Human	ERR1616341
H7	KP02	Netherlands	2016	Human	ERR1616345
H8	KP03	Netherlands	2016	Human	ERR1616353
H9	KP04	Netherlands	2016	Human	ERR1616359
H10	KP05	Netherlands	2016	Human	ERR1616362
H11	H150660738	UK	2016	Human	Unknown
H12	H153900402	UK	2016	Human	Unknown
H13	H135060621	UK	2016	Human	Unknown
H14	H140240552	UK	2016	Human	Unknown
H15	H142140857	UK	2016	Human	Unknown
H16	Kp_1093127	Italy	2016	Human	Unknown
H17	Kp_811117	Italy	2016	Human	Unknown
H18	Kp002	Australia	2016	Human	SRR867655
H19	Kp1832	USA	2016	Human	SRR1222430
H20	38941	Kenya	2017	Human	ERS214375
H21	33909	Kenya	2017	Human	ERS214297
H22	45441	Kenya	2017	Human	ERS219172
H23	NCSR101	Vietnam	2014	Human	ERR025479
H24	DM23092/04	Singapore	2014	Human	ERR025540
H25	DU38032/05	Singapore	2014	Human	ERR025541
H26	SA25	France	2013	Human	Unknown
H27	SA26	France	2013	Human	Unknown
H28	T6	France	2013	Human	Unknown
H29	L3	France	2013	Human	Unknown
H30	Pus_13542	Laos	2015	Human	ERS011870
H31	Pus_15007	Laos	2015	Human	ERS011871
H32	Pus_15987	Laos	2015	Human	ERS011873
H33	NN61	Spain	2009	Human	Unknown
H34	N66	Spain	2009	Human	Unknown
H35	I5	Spain	2009	Human	Unknown
E1	SKP000753	USA	2006	Bovine	ERR025575
E2	SKP000754	USA	2006	Bovine	ERR025536
E3	SKP000755	USA	2006	Bovine	ERR025588
E4	SKP000764	USA	2005	Bovine	ERR025131
E5	SKP000765	USA	2005	Bovine	ERR025132
E6	SKP000827	USA	2007	Bovine	ERR025615
E7	SKP000832	USA	2007	Bovine	ERR025618
E8	SKP000781	USA	2005	Environmental	ERR025151

E9	SKP000826	USA	2005	Environmental	ERR025614
E10	SKP000830	USA	2005	Environmental	ERR025607
E11	ESBLH238T	India	2014	Environmental	Unknown
E12	E48T	India	2014	Environmental	Unknown
E13	19SK1	India	2014	Environmental	Unknown
E14	ESBLH239T	Thailand	2014	Environmental	Unknown
E15	ABW S20	Switzerland	2015	Environmental	Unknown
E16	002 SK5	Switzerland	2016	Environmental	Unknown
E17	2-2	The Netherlands	Unknown	Environmental	Unknown
E18	2-3	The Netherlands	Unknown	Environmental	Unknown
E19	3-1	The Netherlands	Unknown	Environmental	Unknown
E20	3-2	The Netherlands	Unknown	Environmental	Unknown
E21	5-1	The Netherlands	Unknown	Environmental	Unknown
E22	808	Libya	2009	Environmental	Unknown
E23	809	Libya	2009	Environmental	Unknown
E24	817	Libya	2008	Environmental	Unknown
E25	DR85_08	Singapore	2008	Environmental	ERS011922

Table S6 Specific background information of the *K. pneumoniae* strains.

Strain	Source	Cow number	Year
HLJ-1	Farm A	140142	2021
HLJ-2	Farm A	140439	2021
HLJ-3	Farm A	141011	2021
HLJ-4	Farm A	150170	2021
HLJ-5	Farm A	151054	2021
HLJ-6	Farm A	160427	2021
HLJ-10	Farm A	161158	2021
HLJ-14	Farm A	170581	2021
HLJ-16	Farm A	170872	2021
HLJ-20	Farm A	180218	2021
HLJ-23	Farm A	181270	2021
HLJ-24	Farm A	190281	2021
HLJ-24F	Farm A	190281	2021
SD-1	Farm B	158069	2021
SD-3	Farm B	174827	2021
SD-6	Farm B	183568	2021
SD-7	Farm B	181566	2021
SD-8	Farm B	175611	2021
SD-9	Farm B	140361	2021
SD-10	Farm B	131890	2021
SD-13	Farm B	193586	2021
SD-14	Farm B	193189	2021
SD-15	Farm B	175239	2021
SD-16	Farm B	167058	2021
SD-18	Farm B	171431	2021
SD-19	Farm B	172361	2021
SD-20	Farm B	145156	2021
SD-21	Farm B	184381	2021
SD-23	Farm B	172943	2021
SD-25	Farm B	173540	2021
SD (2)-7	Farm B	180653	2021
AH-1	Farm C	155500	2021
AH-5	Farm C	175145	2021
AH-12	Farm C	168544	2021
AH-13	Farm C	14701	2021
AH-16	Farm C	187972	2021
AH-17	Farm C	178388	2021
AH-20	Farm C	152870	2021
AH-21	Farm C	193067	2021
AH-23	Farm C	164864	2021
JS-1	Farm D	4068	2021
JS-2	Farm D	6295	2021

JS-5	Farm D	7254	2021
JS-10	Farm D	2386	2021
JS-11	Farm D	7392	2021
WH-1	Farm E	170593	2021
WH-3	Farm E	180195	2021
WH-5	Farm E	180135	2021
WH-6	Farm E	170520	2021
WH-10	Farm E	180252	2021
WH-11	Farm E	170225	2021
WH-13	Farm E	180359	2021
HB-2	Farm F	159468	2021
HB-3	Farm F	152981	2021
HB-6	Farm F	172756	2021
HB-8	Farm F	822952	2021
HB-11	Farm F	184304	2021
HB-16	Farm F	159067	2021
HB-17	Farm F	183979	2021
HB-20	Farm F	161668	2021
HB-21	Farm F	164740	2021
HB-22	Farm F	188287	2021
HB-24	Farm F	188235	2021
NX-11	Farm G	16483	2021
NX-16	Farm G	18759	2021

Table S7 Detection rates of *K. pneumoniae* in different farms.

**Dairy farm**	**Sampling time**	**Sample quantity**	**Positive sample quantity**	**Detection rate (%)**
Farm A	2021.07	24	13	54.17
Farm E	2021.07	13	7	53.85
Farm B	2021.08	38	18	47.37
Farm F	2021.09	24	11	45.83
Farm G	2021.09	17	2	11.76
Farm D	2021.10	12	5	41.67
Farm H	2021.10	25	0	0
Farm C	2021.10	28	9	32.14

Table S8 Results of antimicrobial susceptibility testing.

Types of antibiotics	Antibiotics	S		I		R	
		Strain number	Proportion (%)	Strain number	Proportion (%)	Strain number	Proportion (%)
β-lactam/β-lactamase-inhibitor combinations	Penicillin G	0	0	0	0	65	100
	Ampicillin	0	0	1	1.54	64	98.46
	Amoxicillin/clavulanic acid	0	0	0	0	65	100
	Ceftiofur	17	26.15	14	21.54	34	52.31
Fluoroquinolones	Enrofloxacin	60	92.31	4	6.15	1	1.54
	Sulfamethoxazole	0	0	1	1.54	64	98.46
Aminoglycosides	Spectinomycin	4	6.15	23	35.39	38	58.46
	Tobramycin	49	75.39	5	7.69	11	16.92
Cephamycins	Cefoxitin	48	73.84	9	13.85	8	12.31
Chloramphenicols	Florfenicol	9	13.85	15	23.08	41	63.07
Macrolides	Erythromycin	0	0	0	0	65	100
Tetracyclines	Doxycycline	19	29.23	8	12.31	38	58.46

Table S9 Statistics of Nanopore data quality.

Strain	Sequence number	sum_len (bp)	min_len (bp)	avg_len (bp)	max_len (bp)	N50 (bp)
SD-14	58 821	1 000 008 661	7 533	17 000.9	134 766	19 434
HB-21	54 597	1 000 019 433	9,785	18 316.4	91 271	19 533

Table S10 The genomics islands prediction of *K. pneumoniae* strain SD-14.

GIs ID	Sequence ID	Start	End	GIs length (bp)
GI1	assembly_1	1 165 213	1 185 458	20 246
GI2	assembly_1	2 049 012	2 081 938	32 927
GI3	assembly_1	2 277 317	2 293 045	15 729
GI4	assembly_1	2 879 024	2 923 750	44 727
GI5	assembly_1	2 958 582	2 991 994	33 413
GI6	assembly_1	3 060 808	3 071 120	10 313
GI7	assembly_1	3 221 868	3 268 487	46 620
GI8	assembly_1	4 669 391	4 696 362	26 972
GI9	assembly_1	4 887 309	4 895 520	8 212
GI10	assembly_2	30 386	48 614	18 229
GI11	assembly_2	103 225	118 189	14 965

Table S11 The genomics islands prediction of *K. pneumoniae* strain HB-21.

GIs ID	Sequence ID	Start	End	GIs length (bp)
GI1	assembly_1	756 743	785 165	28 423
GI2	assembly_1	1 461 283	1 515 264	53 982
GI3	assembly_1	2 962 544	2 993 406	30 863
GI4	assembly_1	2 999 915	3 025 821	25 907
GI5	assembly_1	3 179 377	3 234 127	54 751
GI6	assembly_1	4 205 438	4 220 159	14 722
GI7	assembly_1	4 654 342	4 662 352	8 011
GI8	assembly_1	4 667 975	4 692 945	24 971
GI9	assembly_2	99 711	107 987	8 277
GI10	assembly_2	122 655	128 859	6 205
GI11	assembly_2	235 824	245 742	9 919
GI12	assembly_3	9 964	18 942	8 979

Table S12 The values of alleles and the respective sequence types of 104 strains.

	Alleles							
Strain	*gapA*	*infB*	*mdh*	*pgi*	*phoE*	*rpoB*	*tonB*	ST
SD-14	2	6	1	5	11	1	15	43
HB-21	2	9	2	1	13	1	38	896
M1	3	1	1	1	3	3	1	4
M2	10	20	2	1	9	11	14	442
M3	2	1	2	17	27	1	39	107
M4	2	1	99	6	1	1	129	867
M5	18	22	26	63	85	20	51	414
M6	4	3	1	1	43	1	13	191
M7	2	3	2	2	6	4	4	29
M8	2	3	6	1	9	7	299	5734
M9	4	3	1	1	43	1	13	191
M10	2	1	1	37	3	4	64	1496
M11	2	1	2	1	3	4	4	109
M12	2	1	2	17	27	1	39	107
M13	2	1	5	1	17	4	42	111
M14	10	20	2	1	9	11	14	442
M15	4	7	1	37	177	4	6	2159
M16	2	1	2	1	7	1	24	234
M17	3	5	1	1	12	4	46	1426
M18	4	5	2	2	1	1	24	2253
M19	2	1	1	1	10	1	9	5753
M20	2	1	97	1	9	4	13	846
M21	38	19	53	58	73	21	130	526
M22	3	1	1	1	20	2	62	5754
M23	4	1	2	6	2	5	9	5755

M24	4	6	1	1	8	1	56	229
M25	2	9	2	1	13	1	10	309
M26	4	3	1	36	9	10	14	661
M27	2	1	2	2	10	4	19	1117
M28	2	20	2	1	9	11	355	5759
M29	4	1	1	1	21	1	35	76
M30	17	19	28	20	103	18	52	5760
M31	17	19	92	39	170	18	125	3212
M32	2	7	2	1	61	1	4	3664
M33	4	3	1	36	9	10	14	661
M34	2	1	2	1	2	1	2	776
M35	2	1	5	1	17	4	42	111
M36	2	1	2	3	27	1	39	219
M37	2	9	2	1	13	1	16	37
M38	2	1	1	1	7	1	12	485
M39	3	1	2	1	3	4	31	1086
M40	2	5	2	2	7	1	10	48
M41	5	1	5	1	7	1	24	87
M42	2	5	2	2	7	1	10	48
H1	16	3	2	1	1	1	18	769
H2	11	3	2	2	6	4	4	754
H3	17	19	39	39	51	18	72	196
H4	10	1	1	1	12	1	38	225
H5	10	7	2	2	3	25	4	247
H6	2	5	2	2	7	1	10	48
H7	10	1	2	1	9	27	6	359
H8	14	1	2	1	7	4	182	873
H9	2	1	2	1	2	1	4	1836
H10	4	1	11	1	9	4	59	1593
H11	2	7	2	1	2	1	2	2201
H12	2	3	65	6	3	15	4	2202
H13	2	6	1	37	3	27	111	2203
H14	10	1	11	1	9	10	300	2204
H15	3	31	2	1	4	1	220	2205
H16	2	6	88	5	4	1	6	2287
H17	16	18	21	33	55	59	75	2288
H18	2	6	1	5	4	1	6	101
H19	2	6	1	3	8	1	15	42
H20	3	1	5	1	1	4	223	2813
H21	4	1	2	1	1	1	247	2814
H22	4	2	2	1	247	4	25	2815
H23	3	3	1	1	1	1	4	11
H24	3	3	1	1	1	1	4	11
H25	3	3	1	1	1	1	4	11

H26	2	1	1	1	1	4	19	380
H27	2	1	1	1	1	4	19	380
H28	43	1	2	1	10	4	13	375
H29	2	1	1	1	10	4	13	25
H30	16	24	43	38	54	22	346	2139
H31	12	1	1	2	5	1	36	133
H32	2	1	1	1	9	4	12	23
H33	2	3	1	1	10	1	19	13
H34	3	1	2	1	1	1	4	134
H35	1	6	1	1	1	1	1	14
E1	2	9	2	30	13	1	16	177
E2	2	1	2	2	7	4	4	222
E3	2	1	2	1	13	1	16	256
E4	23	31	2	1	9	4	23	187
E5	2	1	2	6	9	1	10	224
E6	4	5	1	1	12	4	46	289
E7	3	37	1	1	10	4	4	293
E8	2	5	1	6	9	1	10	221
E9	2	1	2	42	26	4	18	211
E10	2	1	2	1	13	1	38	230
E11	4	1	1	1	7	1	1	1739
E12	17	19	39	20	150	18	52	1740
E13	2	3	2	1	17	4	42	1741
E14	2	3	1	1	220	1	4	1743
E15	65	110	189	1	260	4	170	2256
E16	18	19	175	114	261	21	338	2257
E17	2	1	2	1	1	1	25	63
E18	12	1	1	2	23	1	36	50
E19	4	5	1	1	9	1	31	46
E20	2	1	15	1	18	4	30	62
E21	2	1	1	14	24	1	19	52
E22	38	19	69	39	96	51	126	509
E23	50	19	66	20	97	18	126	486
E24	2	1	2	1	1	1	112	511
E25	18	15	26	22	94	13	165	734

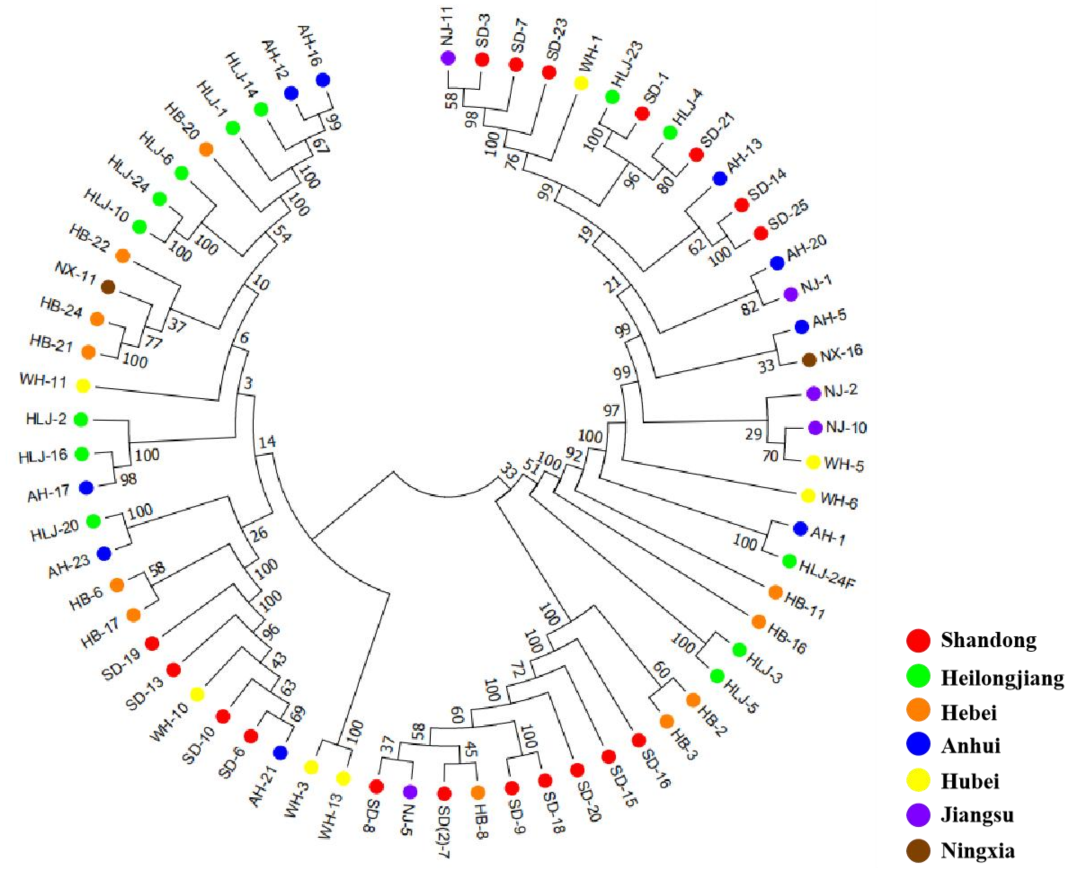

Figure S1 Phylogenetic tree of 65 strains of *K. pneumoniae*

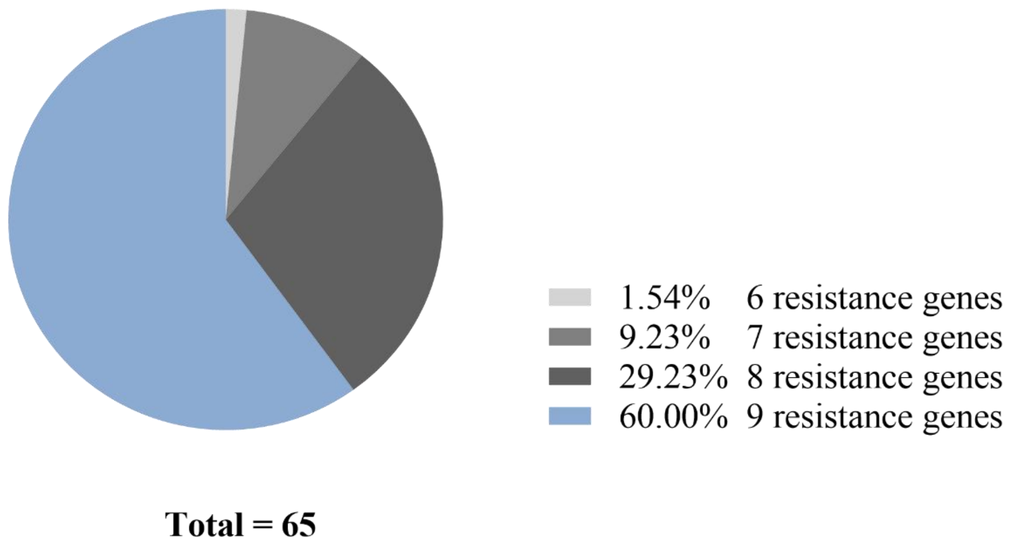

Figure S2 Carriage rate of multiple antimicrobial resistance genes.

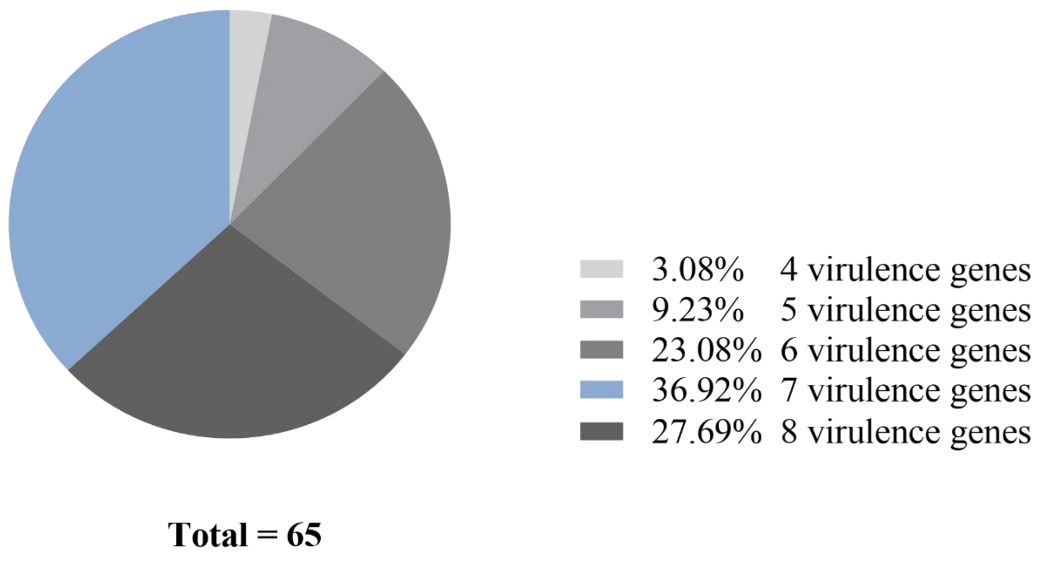

Figure S3 Carriage rate of multiple virulence genes.

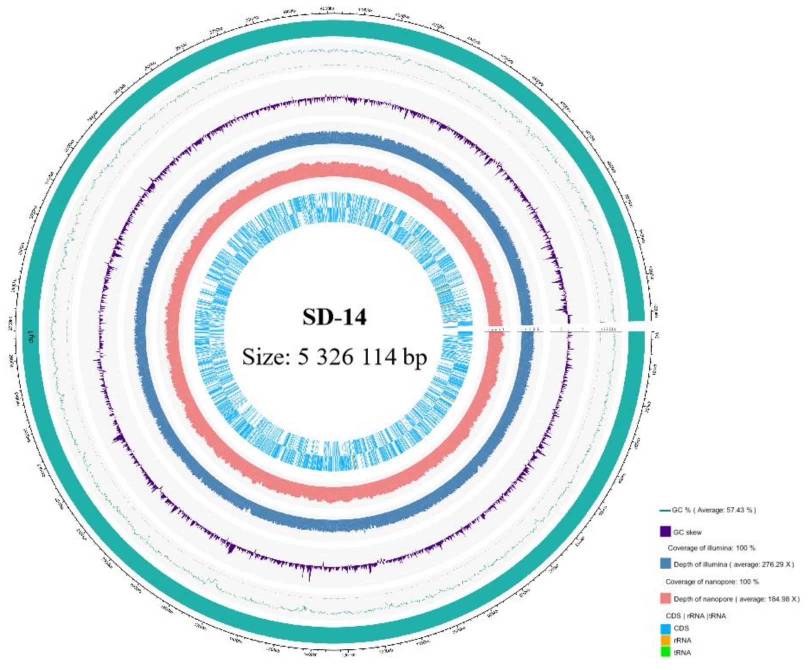

Figure S4 Circular map of *K. pneumoniae* strain SD-14. From the outside to the inside: the first ring corresponds to the information of genome sequence； the second ring corresponds to GC content；the third ring corresponds to GC skew；the forth ring corresponds to Illumina sequencing depth；the fifth ring corresponds to Nanopore sequencing depth；the last ring corresponds to CDS and ncRNA.

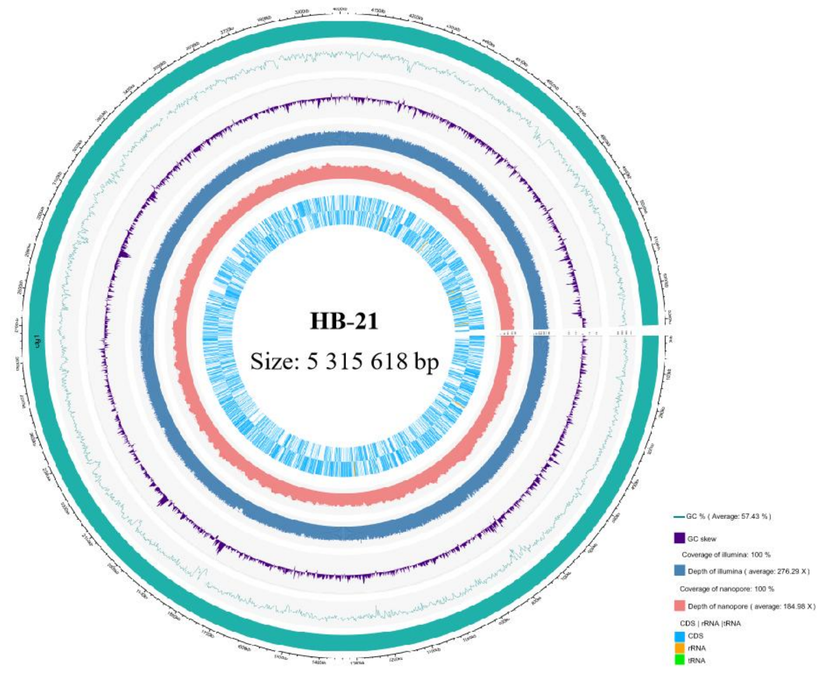

Figure S5 Circular map of *K. pneumoniae* strain HB-21. From the outside to the inside: the first ring corresponds to the information of genome sequence； the second ring corresponds to GC content；the third ring corresponds to GC skew；the forth ring corresponds to Illumina sequencing depth；the fifth ring corresponds to Nanopore sequencing depth；the last ring corresponds to CDS and ncRNA.

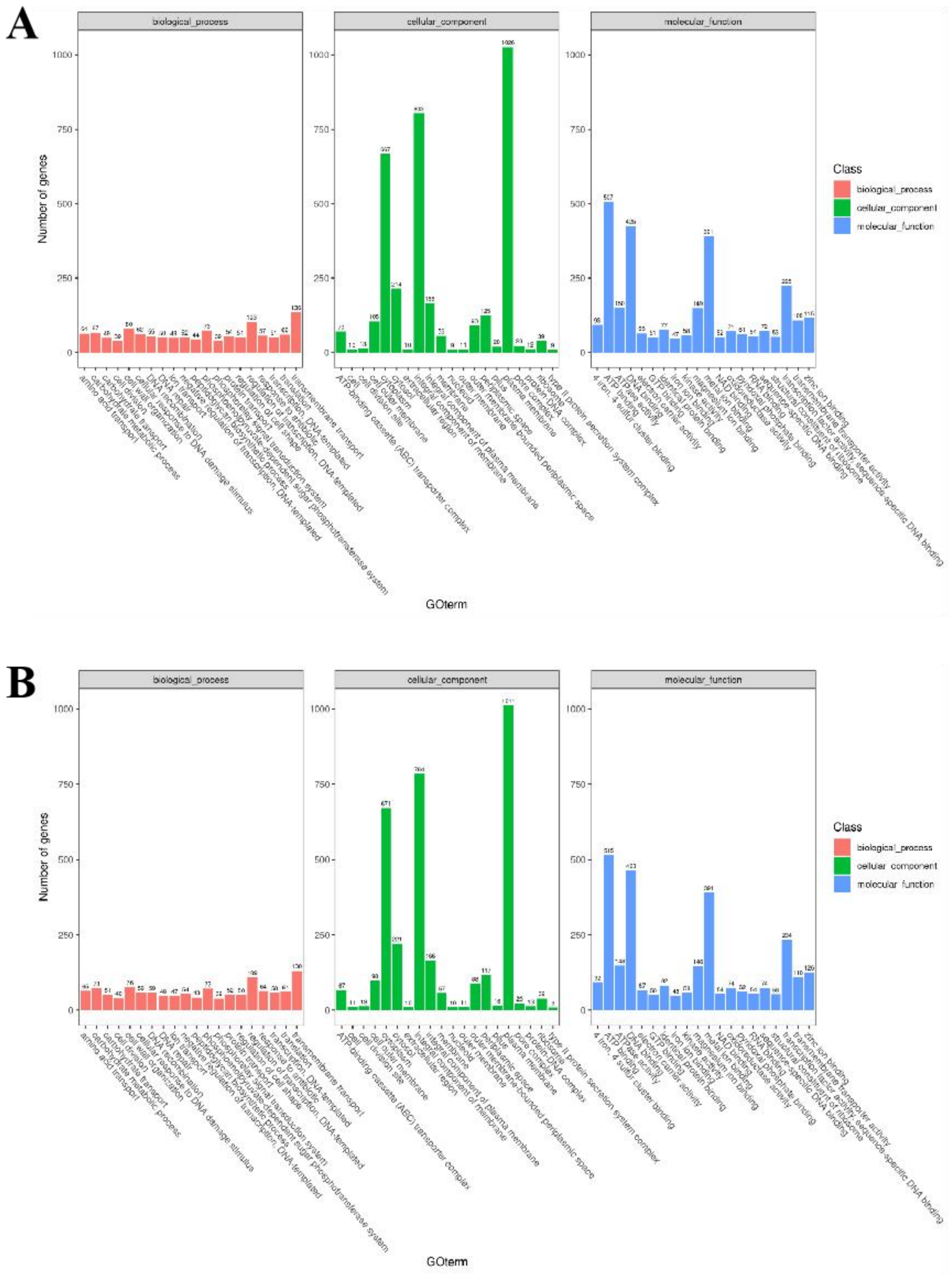

Figure S6 (A) GO classification of *K. pneumoniae* strain SD-14. **(B)** GO classification of *K. pneumoniae* strain HB-21.

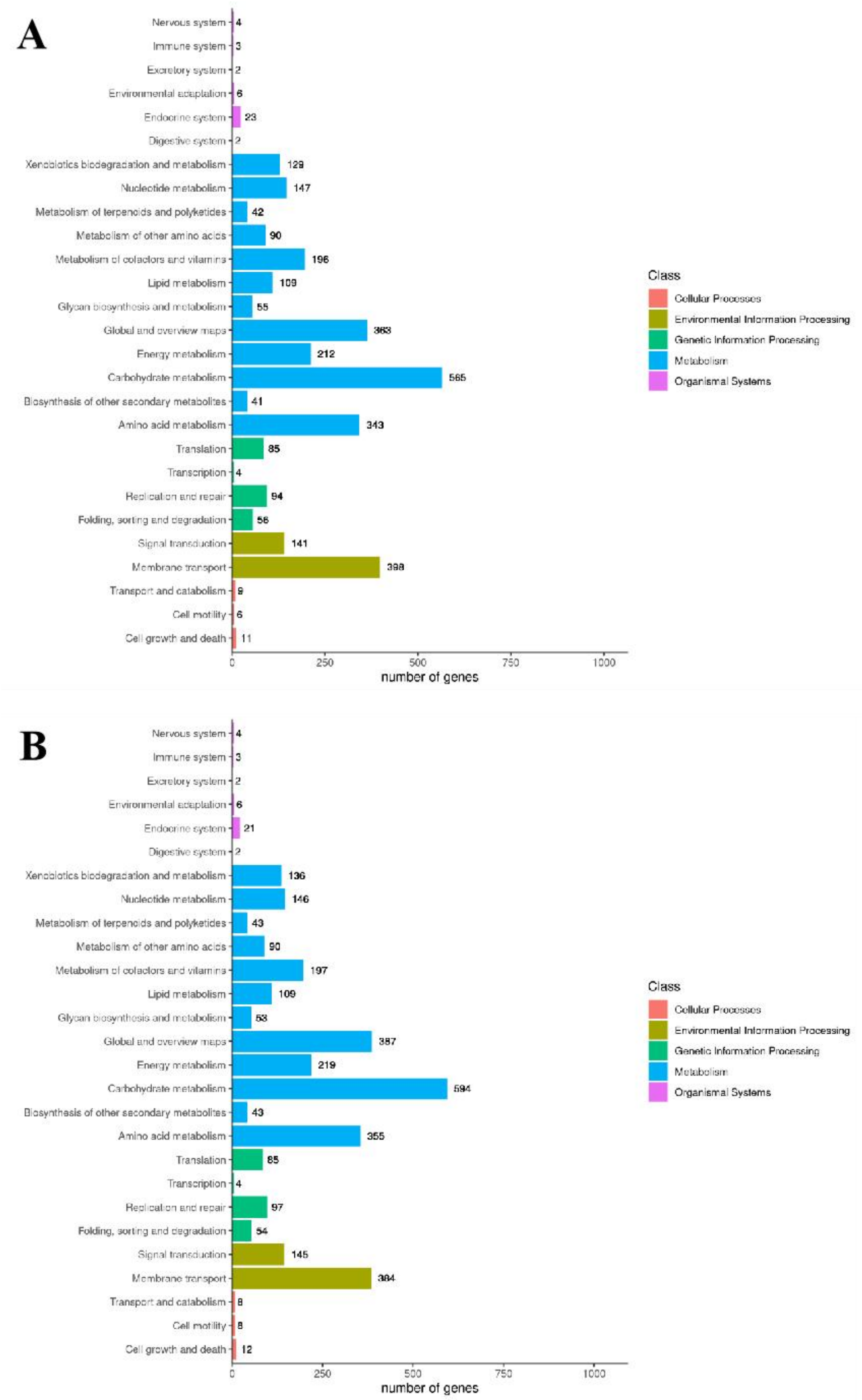

Figure S7 (A) KEGG classification of *K. pneumoniae* strain SD-14. **(B)** KEGG classification of *K. pneumoniae* strain HB-21.
